# A hypoxia research-driven path led to identifying neuroprotection mediators: an interview with Dr. Nicolas G. Bazan

**DOI:** 10.1038/s41420-025-02436-6

**Published:** 2025-04-04

**Authors:** Nicolas G. Bazan

**Affiliations:** Neuroscience Center of Excellence, School of Medicine, Louisiana State University Health New Orleans, New Orleans, LA USA

**Keywords:** Neuroscience, Cell biology

## Introduction

The recollections evoked in this interview are a journey triggered by the insightful review “Hypoxia as a medicine [1],” which made the point that hypoxia has beneficial health consequences. This premise has driven Dr. Nicolas G. Bazan’s research for over six decades [2]. Here, he places in context how his interest in hypoxia was started and connected with biomedical issues.

Dr. Bazan is a “provinciano” born in the Tucuman province of Argentina. He grew up in the province of Salta and was exposed to the changing society in the late 1940s and early 1950s by the workers’ movement seeking social justice, where his father was involved. When the military coup took power, he was 13 years old and witnessed his father start from scratch with a family of three children, reinventing himself and embracing strength, values, and ideals to emerge from the ashes and provide opportunities to his children. Bazan’s parents were devoted practicing Catholics. His mother, a school teacher, went to law school in her late forties and became a JD, practicing law thereafter.

Dr. Bazan’s memories related to medical issues started from a childhood experience, and those related to hypoxia during his time as a medical student were instrumental in his decision to go through a research route. This interview is a glimpse into his work on hypoxia as a backbone, which includes his early times, motivations, departure from Argentina, and life thereafter in New Orleans.

## CDDiscovery: Dr. Bazan, can you remember for our readers your time at medical school and beyond?

As a second-year medical student at the University of Tucuman in northern Argentina (medical school took 6 years; I was 18 years old because my mother made me skip earlier school years), I was unable to find a research project that interested me. So, I went to Buenos Aires (900 miles away) and asked to meet Bernardo Houssay, MD (1947 Nobel Prize in Physiology or Medicine). He attentively listened to me, was very kind, and introduced me to a guest that he happened to have that day, Dr. Hugo Pablo Chiodi. Dr. Chiodi was leading the Institute of High-Altitude Biology in Jujuy, located about 200 miles further north than Tucuman, and he invited me to spend the next summer in his lab. I applied and obtained a summer fellowship from my school to work there.

My fascination with the prospect of my first research experience was enhanced by a study that Chiodi published on a cerebral form of mountain sickness (where the affected subject was himself). I was housed on the institute’s upper floor dorm—a building that had roomed Dr. Salvador Maza, who contributed to the understanding of the Trypanosoma Cruzi disease called Chagas-Maza.

At this time, Dr. Chiodi’s labs were dedicated to unraveling basic issues of hypoxia at high altitude, and he had an ardent dedication, arriving at 7:00 AM and working long hours daily. I followed him closely, learning avidly by his side. Recognizing my passion, he assigned me to a project to thyroidectomize and adrenalectomize rats before exposing them to hypoxia in a high-altitude simulation chamber. One day, I observed a brownish tissue that appeared subcutaneously in the interscapular area, resembling adrenal glands. I stayed overnight and assessed all the animals, finding that this phenomenon only occurred in rats subjected to hypoxia, not in the sham controls. The following day, I asked Dr. Chiodi what the apparently newly formed tissue was, and he did not recognize it but encouraged me to find out what it was. I spent long hours digging through the literature in the institute library (including chemical and biological abstracts in those days), setting up stains for histology, and conducting additional experiments. Thus, the tissue that evolved was white adipose tissue (displaying tightly packed mitochondria with few fat vacuoles) that, upon stimulation by hypoxia, was transformed into brown adipose tissue. Brown adipose tissue is key for mammalian survival in cold environments and stress conditions, and currently, there is a renewed interest in the browning of white adipose tissue in obesity, metabolic syndrome, and diabetes. While in medical school, my early observations were published in an article and a review in Spanish [3, 4].

Shortly after, political upheavals in Argentina forced Dr. Chiodi to emigrate, and he accepted a faculty position at the Department of Physical Medicine and Rehabilitation, P&S, Columbia University, NY, and invited me to be a fellow in his lab. It was my year after medical school. After one year, he moved to Santa Barbara, CA.

During the following summer, after my experience with Dr. Chiodi, I went back to Buenos Aires and was a student fellow at the Luis Leloir Institute (1970 Nobel Laureate) and worked under a great mentor, Dr. Hector Torres. As a medical student, during the year, I volunteered at the Institute of Biology under Profs. Else Brauckmann and Francisco Barbieri and learned about early amphibian development.

## CDDiscovery: What happened when you moved from New York City to Boston?

When at the P&S, Columbia University, I actively searched to work on the nervous system. I wrote to Cliffe D. Joel, Harvard Medical School, because of his work on brown adipose tissue and his interest in brain lipids. The lab was interested in the brain, and Cliffe was a former trainee of Manfred Karnovsky on lipid biochemistry. The intellectual enjoyment was momentous during lab meetings and seminaries. Cliffe facilitated and encouraged me to follow up on an observation that I made on a thin-layer chromatography plate as his postdoc on the effect of hypoxia. Those days, free fatty acids (FFAs) were studied as uncouplers of oxidative phosphorylation from the respiratory chain [5–7].

Our ideas went in a different direction to test if hypoxia modifies brain FFA; their assessment was arduous because the available acid base titration, colorimetry, and column chromatography, were not reliable. I began experimenting by running brain lipid extracts on TLC plates with organic solvent mixtures that let phospholipids remain at the spotting site and separate neutral lipids, where FFA should migrate, in a run. I observed that either overload of the absorbent produces striking and no separation or if the thickness of the absolvent was greater, to attain higher loading capacity, it would not allow us to see trace components like the FFA pool. So, we came up with the idea to use a TLC plate that would overcome the disadvantages of even thickness TLC, either too thin or too thick. The method involves chromatography on a TLC layer of Silica Gel that decreases linearly in thickness from 1,000 μm at the base to 125 μm at the upper end [8].

This design allowed the separation, preparative isolation and densitometric quantitation of small amounts of FFA from relatively large lipid extracts with advantages of specificity, simplicity, rapidity, reproducibility, accuracy, and high sensitivity [8, 9].

In addition to this technical development, in retrospect, I believe that the following observation set apart our initial study on the effect of hypoxia on brain FFAs. We observed that the faster I sampled the rat brain by rapid decapitation, dissection, and the start of homogenization in organic solvents, the lower the FFA content. I presented this finding of fast brain FFA release as a response to hypoxia along with the new gradient thickness TLC method at FASEB 1968 [9]. Our paper was rehearsed at the Department of Biological Chemistry, Harvard Medical School, receiving praise from Jordi Folch and others. The period in Cliffe laboratory was important as a postdoctoral fellow in 1966, when I was 24 years old. However, Cliffe shared with me his uncertainties in many facets of his life, particularly his depressive swings, and just one year later, he decided to move to Lawrence University, Appleton, Wisconsin, primarily to teach, and I was promoted to an instructor position and left in charge of the lab. Due to a visa issue, I decided to explore jobs in other countries.

## CDDiscovery: Please tell us more about your early childhood experience and motivation

Gabriel Garcia Marquez said in one of his latest books, “Life is what we remember and how we remember it.” The beginning of my journey was the decision to go to medical school, likely impacted by an experience that I underwent when I was eight years old. The following is how I describe my reminiscence of this event as Dr. Alvaro Cruz [in my novel *Una Vida* [10]]:

“Cruz believed that human brains were built for the urgent program of ongoing re-creation that works like a spark during successful aging. In others, the spark unaccountably failed. In his research, Cruz felt closer than ever before to identifying the triggering keys that failed to work in ‘unsuccessful aging’ of the brain. At eight, the course of his life was set when he witnessed his aunt having a grand mal seizure in Salta, when she was taking him to his piano teacher’s house. His aunt began to convulse violently, then fell with a thud onto the muddy cobblestone street. Young Cruz watched in fascinated horror, as though from a great distance that grew greater each moment, as her body flailed like a chicken whose neck had just been wrung. Her feet were shaking as though someone were choking her; she was clawing for her life. Cruz had heard about demons from the priests. An invisible one must be strangling Tia Tita, he decided, despite her having just made the respectful sign of a cross as they passed the church. The butcher ran out of his shop with bloodied hands. He grabbed hold of Tia Tita and tried to subdue her, getting blood all over her golden dress. It looked as though she were being murdered. People gathered around as the butcher forced a spoon into her mouth to keep her from swallowing her tongue. Cruz remembered wondering why anyone would swallow their own tongue. In those days, children learned only as much as their parents thought they needed to know, so Cruz had never been told about his aunt’s condition. His mother told him afterward that her sister has epilepsy, a brain disease. But there, in the sweltering street, he somehow understood that the brain held all the power. It could make things happen that no part of the conscious self could control. In throwing his fastidious Aunt Tita to the street, her brain made it clear that it could exert its own agenda whenever it desired. That gargantuan, mysterious, arcane entity that had a hand in every aspect of life became the sole focus of Cruz’s curiosity from that moment on, the only riddle worthy of his questioning mind. His single, infinitely complex case to crack. From that day on, the questions of the brain’s function, origin, outer limits, and especially ability to turn on the body—its proprietor—haunted, intrigued, bewildered, startled, and inspired Alvaro Cruz. It did all those things, but it also focused him on his own insufficiency, his own impotence—a condition caused by loving his aunt too much. If he had not loved her, he might have cried out in time. The shock of her brain’s interference in their love was what had triggered the distancing, he believed. He had resolved that day to become a detective of the brain and to take absolutely nothing for granted. Not even God, who, at that moment, had apparently been looking the other way. Cruz had learned that not only was the brain a worthy adversary, but it was also an immensely complex labyrinth of riddles and crenulations. He was convinced that in the charting of the brain’s undulating, ever-changing maze, he would find the key to the overriding secret at the core of it all. He thought of the short poem by Robert Frost: ‘We dance round in a ring and suppose. / But the Secret sits in the middle, and knows.’ The ‘middle’ was the synaptic gap. The human brain is a creative organ—it interprets events, not just records them. It functions more like an abstract painter than a realist. Its view of what happens is more important than what happened. Pain, smell, taste, touch, and fear are the visionaries of what we see. There is no pure, unmediated seer; no objective reporter. Our images are shaped by the context in which we choose to live. We see what we want to see, hear what we want to hear—or what we need to preserve the image of ourselves and others that provide us with a sense of purpose.

“A seizure like Aunt Tita’s rapidly depleted the brain of energy-providing chemical fuel. Cruz had been fascinated by how that complexity was coordinated so harmoniously, and he’d experimentally tackled the question of how endogenous chemicals might prevent brain demise when adverse conditions arose. Seizures had been a recurring focus of his research, and only a few years ago, he’d connected the dots with that early childhood experience. Stroke, age-related macular degeneration (AMD), retinitis pigmentosa, epilepsy, Parkinson’s, and AD all represent conditions that reflect an intrinsic biochemical signaling failure—like a conductor facing his orchestra after having forgotten the score.

“The conundrum that had been occupying Cruz’s mind that morning was *identity*. How did the ocean of trillions of separate neurons, with billions of points of synaptic connections, hold together a single unified self—a person—with a unique mind of his or her own? Was the patient with Alzheimer’s still “himself?” Cruz knew he had to reexamine his answer to that question, or the sorrow would never go away. Though most neuroscientists made approximations on how the binding of a self was done, in general, they relegated such speculation to the bailiwick of the philosopher. In today’s neuroscience, specialization is an accepted norm and duty. The brain as a whole was simply too vast for any one researcher or one laboratory.

“Cruz, trying to never lose sight of the whole, had played the game of specializing in the parts. Much of his research at the Center was focused on trying to understand how essential brain lipids contributed to the survival of cells in experimental models of retinal degeneration, stroke, epilepsy, and Alzheimer’s. He thought that the brain may have fundamental signaling to support its organization and that various diseases affected similar events. The failure to counteract such destructive forces gave rise to disease states with loss of brain cells. The keys were in those synaptic portals—they had to be—yet all too many of them remained locked.”

## CDDiscovery: We learned that your first laboratory was in Toronto, Canada

At FASEB 1968, Leon Wolfe from McGill University, Montreal, and Roger Rossiter from London, Ontario, who were leaders of lipid neurochemistry, were at my presentation. Rossiter invited me to give a seminar and offered me a position in his lab, and Leon facilitated an interview in Toronto, where in 1968, I became Assistant Director, Department of Neurochemistry, Clarke Institute of Psychiatry, jointly appointed Assistant Professor, Department of Biochemistry, Faculty of Medicine, University of Toronto, Canada, where I set up my first laboratory. I decided first to ask which brain-specific FFAs were released so rapidly during hypoxia by post-decapitation ischemia. So far, we only knew that the total pool was enhanced [8]. So, we adopted preparative gradient thickness-TLC, methanolysis and GLC (flame ionization detector) and found that docosahexaenoic acid (DHA) and arachidonic acid (AA) were released and that their rate of release was similar to that during maximal hormonal lipolytic stimulation [11]. So, a question was whether this event was a postmortem phenomenon, and which was the source. My lab was at the Clark Institute of Psychiatry, where I learned that electroconvulsive therapy was used for depressive illness. I thought that by applying ECS to rats, extensive synaptic stimulation would be activated, not causing brain damage, in a reversible fashion. A bright medical student, Harry Rakowski, during a summer project, showed that, in fact, brain FFA accumulated in seconds in a reversible manner [12]. Then, we demonstrated that as hypoxic ischemia did, ECS promoted the transient accumulation of the FFAs. Looking at the source and mechanism of the event, we observed that the brain triglyceride pool is small and did not change in ischemia or after ECS, indicating that a triglyceride lipase was not involved. We suggested that a phospholipase A2 (PLA2) ought to be activated and were able to show the activities of PLA2 and PLA1 in brain subcellular fractions [11, 13].

During this time, beneficial health effects of diets rich in omega-3 fatty acids became apparent [14–17]. Thus, we began conceptualizing and pondering the biology of the omega-3 family member DHA, which is prominently concentrated in the central nervous system (CNS), and we formulated hypotheses and tested them under various hypoxic conditions and others, as described below.

Medical sciences in the 1960s were captivated by the discovery of prostaglandins from AA [18]. We took a different approach, aiming to understand the effect of hypoxia and the consequences of brain DHA release. Our initial paper [11] became a Citation Classic [19] by showing that DHA and arachidonic acid are released during seizures or hypoxia during ischemia by PLA2. Then, many outcomes evolved, including our finding that a related lipid, the phospholipid-mediator, platelet-activating factor (PAF), modulates hippocampal excitatory synaptic transmission, presynaptic glutamate release and is a retrograde messenger of LTP enhancing memory formation [20–22]. PAF synthesis involves DHA and AA release. Thus, we began connecting these findings with synaptic signaling, function, and neuroinflammatory responses.

## CDDiscovery: Why did you decide to return to Argentina?

The science in our Toronto lab flourished, and it was well-funded. However, my recurrent dream was to do what I was doing in Canada in my own homeland. I had mentioned this to my friend, Esteban Brignole, with whom I connected with when he was a chemical engineer fellow at MIT and I was at Harvard Medical School. At the time, Dr. Brignole was strengthening academics at the University of the South (UNS) in Argentina. He knew about my dream and desire to one day return to Argentina, excluding Buenos Aires, where most biomedical academics were concentrated. I felt that there was a need for academic/research development in the provinces.

I arrived at the UNS in early 1970 as a 28-year-old dreamer to establish a new research institute with a focus on fundamental biochemistry to understand disease mechanisms, particularly of the nervous system, including the retina. I also thought that the time was ripe to commence motivating translational bridges to medicine and other Argentine resources like the food industry. We began a conversation with leaders and scientists about the intrinsic worth of high-quality science, education, technological innovations and knowledge-based economic development. That period portrays a story of over a decade with more downturns than upturns due to the oscillating political pendulum of the country and the frequently changing university leadership. The year that I arrived, the University Rector (President) changed three times, and the memories/agreements of the promised startup funds and equipment vanished.

We began a new biology sciences school, a graduate program, and took on the responsibility of teaching biological chemistry and many more chores. In the lab, we began exploring issues of lipids in the brain, retina, and membrane biogenesis using the toad *Bufo arenarum, Hensel*, which were inexpensive and easy to manipulate experimentally. I learned about toads when, as a medical student, I volunteered at the Institute of Biology, University of Tucuman, using early stages of toad development with Else Brauckmann [23] and Francisco Barbieri.

With my first graduate student, Marta Aveldaño, we did many firsts on hypoxia. She and I went around 4 AM to slaughterhouses, which allowed us to get bovine retinas. A key goal was to sample the retinas as soon as possible after death because we hypothesized that hypoxia was a key trigger of the release of FFAs. An integral part of the central nervous system, the retina is richly endowed with long-chain polyenoic fatty acids. FFAs are a relatively small and labile pool composed of a large proportion of polyunsaturated constituents. Our study reported remarkable features of endogenously produced retina FFAs by hypoxia, showing that they arise from membrane lipids and that a different behavior is apparent in the rate of release of individual FFA from the tissue in vitro [24, 25].

We used toad embryos during the early stages of development to ascertain membrane biogenesis. When lipid precursors were supplied at the time of oogenesis, differences in specific activities of phospholipids were observed. Whereas, during cell cleavage, intracellular redistribution of preformed phospholipids was used for membrane assembly [26–30]. Thus, we suggested that phospholipids for membrane biogenesis during early embryogenesis are derived from a storage site (the yolk platelets) through an active and specific intracellular redistribution process. At the arrival of the phospholipid to the nascent membrane, turnover of the phosphorylase may be necessary to assemble the lipid into the newly formed membrane structure [26–30].

I was also paid a visit by Sir John Gurdon to my Argentine laboratory in 1973, and the conversations we had were a strong positive influence on our research program.

We also began a new academic unit in 1973, the Instituto de Investigaciones Bioquimicas, bringing together the university with the National Research Council (CONICET). Nobel awardee Luis Leloir supported the project. Then graduate students and fellows evolved: Marta Aveldaño, Norma Giusto, Carlos Barassi, Marcos Crupkin, Ana Maria Pechen, Mary Pedicone, Telma Alonso, Haydee Pascual, Monica Ilincheta de Boschero, Susana Pasquare, Victor Marcheselli, Elena Rodrigues de Turco, Luis Politi, and many others. We overcame university political commotions and growing community disturbances and were able to develop innovative science with very scarce resources.

We found a much slower rate of FFA release in the toad as compared with mouse and suggested that this reflects the higher resistance of poikilotherms to hypoxia [2, 24, 31]. Exploring the sources of the FFA released from membranes, we investigated microsomes and rod outer segments from bovine retina by argentation TLC after conversion of the phospholipids to labeled acetyl-diacylglycerols [32, 33]. We uncovered highly unsaturated molecular species that we coined the term “supraenes,” which indicate the presence of more than six double bonds per molecule [26, 27].

DHA was the major component of these species, in combination with other unsaturated fatty acids, predominantly polyunsaturates.

## CDDiscovery: When and how did you finally leave Argentina?

*The following section is an excerpt from my book*, The Dark Madonna [34], *documenting my family’s escape from Argentina in 1981 during a period in which the military dictatorship seized and terrorized the country, censoring information and academic research and committing unspeakable human rights crimes, including torture and disappearing some 10,000 people*.

“It was late, and the vestibule was hot, crowded, and smelled of people who had been forbidden from showering. Cruz himself had not showered in days, and his own stench was overpowering him. His water had been cut off, not because the bills weren’t paid but because the government controlled the water company and was at a point of using whatever means necessary to gnaw at people. There hadn’t been electricity in days either. Keep them in the dark and dirty—it was a government obsessed with making everything and everyone exactly as it was.

“Family, colleagues, students, and friends of Dr. Alvaro Cruz were packed together like quarters in a roll—stacked. Some were crying while others hugged silently, apparently out of tears. They were waiting for the plane that would carry Dr. Cruz, his wife Elvira, and their five children to the United States.

“The family’s bags were few, and Dr. Cruz had managed to bring only a couple of precious boxes of research documents along. He hoped his memory would hold out for the rest, for this was before the days of advanced data storage and international access. Everything was being left behind.

“No, not everything. Dr. Cruz had friends and the respect of his peers. So, they were all heading to a new life, but doing so meant leaving a wonderful old life full of hopes and memories. A language, a culture, a heritage, a full-blown identity that having to leave behind felt the closest thing to death that Alvaro personally had come near since he watched his aunt seize on the street when he was a boy.

“This experience triggered his obsession with the brain and, even more, with the delicate and complex reasons for its dysfunction. He couldn’t even bring his books with him. His science books were one thing; he could replace those in New Orleans, he hoped. But his books from his days as an undergraduate, the part of him that was private and unseen in his daily activities but was marked in these books as a testament to the searcher he was, not just as a scientist, but as a man.

“As a young man, he drank up books on philosophy and religion and wrote his thoughts in the margins of Teilhard de Chardin, Gabriel Marcel, Kierkegaard, Camus, Martin Buber, Chesterton, and Kafka as though he were a rabbinic scholar in the heat of Talmudic debate with them. Cruz loved a good argument, especially when the stakes were existential. And these books were the record of his young striving mind, the mind he could not always make publicly known in his career as a scientist. But a mind that nevertheless was still shaping his spirit and showing up in dreams and in what began to feel like strange waking coincidences that bore strange fruit.

“His everyday adult mind was given over to details, experiments testing the excitement of a new idea with real quantifiable results. But his mind always craved more. Neuroscience was just one aspect of what Alvaro Cruz was, and that’s why the Argentine rulers wanted to kill him. If he would just do his science and shut up about it and continue to give the government-sponsored research a good name, that would be one thing. But he thought, too, as a fully conscious man.

“And Alvaro was unapologetic about this, not from egoistic pride, but from the sense that his personal authenticity was nonnegotiable and there wasn’t much he could do but live it out and take the consequences. In this moment, he wished he had those old books with him; they reminded him of who he was, and they gave him strength, and no matter how internally solid he felt, an external reminder was always welcome.

“As everything was being taken away before his eyes, he clung to his wife, Elvira, and the five children standing before him. The genesis of his authentic journey, the texts that helped him to debate and ultimately claim his convictions such that he could feel empowered to stand up even in the face of fear, would now be a memory. He looked down at his youngest, innocent to everything that was happening save the smell and the tight space, and he wondered if Ana would ever come to know the country of her birth.

“These thoughts passed through Alvaro intimately, and for a moment, the stinking place was sacred, and he felt quiet inside. Moved.

“A man approached suddenly, his eyes fixed on Alvaro as he marched arrogantly with the rulers’ endorsed authority of a military man paid to act, not think. Alvaro was brought back to his physical self in total and felt the instinct of fear rise, the quickening pulse, the feeling of the need to protect, to fight to keep his family safe.

“Were they to have made it this far only to be taken in after all? Was the last turn of events a trick the rulers played—take you as close to the finish line as possible, make you feel safe, and then pounce? The man ignored everyone as he strode forward like the dreaded principal, entering the classroom with a scowl, only worse.

“The children grew silent, even Ana. He stared Alvaro down as he neared, bringing his face so close his breath burned across Alvaro’s cheeks. It was a disgusting smell like none he had ever encountered, and it would be a sensation he would never describe to anyone and that he would never forget. Alvaro would be able to recall that face forever, the pale skin, the patchy beard of stringy black hairs, and the dark eyes that held a terrible familiarity with violence.

“The man’s face twisted. He shouted: “We should have gotten rid of you long ago, professor.” Flecks of spit showered Alvaro’s face, but he held still, not even wiping them off. There was nothing to do; numbness came over him, and the passing of time halted. Behind the man, another figure kept his distance. He was costumed in full military garb, complete with a rifle at his side that looked like a prop in some play that had been acted out throughout history too many times with too many confused men settling for these bit parts.

“The man in his face was an underling, thought Alvaro, and that could either embolden him or restrain him. One never knew with the military anymore. So many others, like Alvaro, had never even made it this far. He thought of colleagues and friends who had disappeared in the middle of the night, sometimes with their entire families, never to be heard from again. Since the coup in 1976, his world had begun to quake, the ground opening beneath it swallowing up everything that was routine—a fault line that day by day had taken away all the years of his life that had been carefully constructed and planned. First, they took away everything he had professionally, and then the threats of death against him and his family began.

“The offer from a medical school in the USA was a lifeline, a blessing for him and his family. He pushed away the thought of what could happen if they didn’t make it out, if they couldn’t get on that plane today. This last week was one breath at a time, and that’s how he kept it going now, just following his breathing, trying with all his effort to keep the panic at bay, to find that authentic, fearless place and respond from it.

“The man finished yelling, breathing heavily in the suffocating heat of the small room, exhausted from his own anger. Alvaro continued to follow his own breath, counting inhales and exhales, willing his eyes to betray nothing, no sign of resistance or fear, nothing for the man to react to. Time seemed to stop. A kind of suspension or stalemate. The man reached into his pocket and dug out a small pouch of tobacco and some papers. He rolled a cigarette carefully, trying to regain his composure. He asked Alvaro if he wanted one, and Alvaro consented, not knowing what a refusal would arouse. The man twisted two cigarettes closed, struck a match, and lit them both in his still twisted mouth, now more from tiredness than anger, inhaled and then passed one to Alvaro. Alvaro took a drag without upsetting his already calm breathing pattern. The smoke simply showed an external picture of what had become an even inhale and exhale. Everything else was fading away; only the breathing remained.

“They smoked without a word, the military man regaining his composure and Alvaro watching the external gauge of his even breath. Elvira looked on in rapt amazement. The other military man stepped forward, grabbed a pouch and papers from his own pocket, and rolled his own. The three of them stood there like friends, smoking after a conversation that had run its course without true winners or losers. These silent five minutes felt like a kind of purgatory; they always would. The cigarettes came down to their ends; the men smoked them until their fingers burned.

“The military men smiled the smile of demons eating your soul, patted Alvaro on the head and turned to leave. “Enjoy America, doctor!” they chimed. “Don’t come back! Next time, it won’t be a cigarette we share together!”

“Finally, both military men were in the distance, and their backs walked on out of earshot and passed the point of turning to look”.

“Alvaro’s breath came spilling out uneven and hurried as though he had been able to control it by some magic that had finally given way as if at once it was midnight, and the chariot held out just long enough before turning back to a pumpkin. He looked to Elvira, surrounded by the children, and met a mixture of fear, shock, and relief in her eyes. She nodded to him, silently acknowledging how close they had come to the end”.

“It may have been moments or hours later that the family boarded the plane. Alvaro remembered nothing of how he had found his way on board, or even the many goodbyes which must have taken place. Time continued to swirl and mutate, compressed into Aristotelian dramatic forms. Was it early morning or early evening? Alvaro and Elvira settled their brood into their seats, urging each child to sleep if they could. The children, having been told of the trip only twenty-four hours before, were confused but subdued. They knew something that couldn’t be explained now was happening. Their parents’ unease, a thing they rarely experienced but had seen often these last months, had silenced any objections to the trip. Sorrow and confusion filled the plane as though the cabin was being pressurized by it rather than oxygen. The stories behind the sorrow re-circulated from Argentine family to family, each breathing in the pain of the others”.

“Patricia, the oldest child at fourteen years, watched the only home she’d ever known disappear under a thick blanket of clouds that pulled a cover-up and over the world below. Higher and higher they rose until the window showed nothing but deep blue, and Argentina was a vanished memory absent from whatever was present now.

“Alvaro breathed deeply, letting the relief of escape settle into his bones. The miracle of their good fortune weighed on him simultaneously with their loss. This ambiguity of feeling would be an almost constant refrain now that exile had come to pass. Many were unable to fly away from the open sore that was once his beloved country; he thought of them being swallowed up by it just as he flew over the pampas and the tears ran down his face, the tears he had been holding in for months as he practically dealt with the chaos coming at him daily. His children knew enough to look away.”

“Feeling profound gratitude for his children’s safety and that of Elvira, he also ached at the thought that he might not see his homeland again for a long time and so many other family and friends. Elvira reached for his hand, and he took it as a lifeline to everything he cared about. She was filled with the same mix of joy and sorrow, and the tears streaming down her tired face reminded him to finally wipe his own.”

## CDDiscovery: When did you move to La Nouvelle Orleans?

Appointed faculty at LSU School of Medicine, New Orleans, in 1981, we began the Neuroscience Center of Excellence in 1989 as a multidisciplinary academic unit. DHA is avidly retained in the CNS, where it attains the highest concentrations in the human body. While studying earlier hypoxia-induced DHA release in the brain, we began using the retina as a nature-made brain slice because its differentiated neurons, the photoreceptor cells, are enriched in DHA, and its neuronal circuitry makes it an integral part of the CNS. We stumbled on new mechanisms regarding how DHA is acquired to reach such a unique endowment in the CNS. Thus, we identified the liver-to-brain (and liver-to-retina) “long loop” for DHA supply [35] and a retinal pigment epithelium (RPE)/photoreceptors intercellular “short loop” for DHA retention in photoreceptors [36, 37]. We postulated that this recycling is critical to sustaining DHA in membranes because it configures membrane structure and is also a precursor of bioactive mediators for photoreceptor survival; hence, its disruption leads to retinal degenerations. We found that Usher’s Syndrome patients (dual sensorial neurodegenerative disease, blindness and deafness) have a DHA shortage in the blood, implicating the long loop in retinal degenerations [38].

The collaboration with the insightful Dennis Rice led to the discovery of molecular principles involved in the retention/conservation of DHA necessary for cell survival signaling in RPE cells and photoreceptors. We found a specific transmembrane protein (adiponectin receptor 1; AdipoR1) for DHA uptake/retention in RPE cells and photoreceptors necessary for cell functional integrity [39]. Although this AdipoR1-protein has seven transmembrane domains, it is not a G-protein, and thus, we demonstrated that its cognate ligand, adiponectin, is not involved. Therefore, the new function is that AdipoR1 represents a key molecular switch for DHA uptake, retention, and conversion into a photoreceptor-specific molecular species of PC that is decreased in age-related macular degeneration (AMD). In fact, when the protein was genetically ablated, retinal degeneration ensued [39]. A mutation in AdipoR1 causes nonsyndromic autosomal dominant retinitis pigmentosa, a finding that was based on our work on AdipoR1 [40]. In addition, we identified a second protein, MFRP, as necessary for DHA uptake and to sustain transcriptome architecture for photoreceptor function [41]. MFRP deletion (*MFRP*^*rd6*^) leads to photoreceptor cell degeneration.

## From fundamental hypoxia research to neuroprotective mediators

Cells activate responses to sustain function/s at disturbance onset that could result in damage. The concept upon which our experimental search took place was based on the idea that the nervous system, at the onset of hypoxia, defends itself by activating the production of molecular guardians to preserve cellular integrity. We have used two experimental models to test the prediction that pro-homeostatic neuroprotective mediators, to counteract damage disruptions, would be generated: hypoxia in ischemia-reperfusion in stroke and photoreceptor degeneration. Moreover, we further tested this expectation in a human disease—age-related macular degeneration (AMD)—and in other conditions described here. In the search for protective guardian(s), we initially uncovered that brief hypoxia triggered the release of free docosahexaenoic acid (DHA). Subsequent studies led us to identify the DHA-derived lipid mediators—neuroprotectin D1 (NPD1) and elovanoids (ELVs). These are low-abundance, highly potent mediators that elicit neuronal survival and counteract cell damage by oligomeric amyloid-beta (OAβ) peptides in experimental ischemic stroke, epileptogenesis, traumatic brain injury (TBI), retinal degenerations, and other neurodegenerative diseases. Furthermore, ELVs contribute to resilience by reshaping the transcriptome architecture, enhancing the abundance of homeostatic proteins, reducing proteins associated with cell damage, and modulating the activity of thioredoxin reductase 1 (TXNRD1) in uncompensated oxidative stress. Thus, lipid mediators, the formation of which is activated by brief hypoxia and onset of cell damage, foster homeostasis, a redox signaling environment; downregulate genes associated with senescence, autophagy, extracellular matrix remodeling, inflammaging; and elicit neuronal integrity.

Following the early observation of hypoxia-mediated release of DHA (as in brain ischemia), we demonstrated that DHA induces cell survival in ischemic stroke [42, 43], modulates neuroinflammation, and activates long-term restoration of synaptic circuits in models of epileptogenesis [44, 45]. Thus, DHA released by hypoxia has well-defined beneficial effects. We developed the concept of bioactive DHA derivatives by calling them docosanoids (22 C, in contrast to the 20 C eicosanoids from AA) [46]. Thus, we demonstrated that DHA bioactivity is elicited through its conversion into docosanoids, which halt the generation of cell death signals.

We experimentally designed an early post-hypoxia condition that allowed us to find the synthesis and bioactivity of neuroprotectin D1 (NPD1; 10 R,17S-dihydroxy-docosa4Z,7Z,11E,13E,15Z,19Z-hexaenoic acid) [47] and coined its name [48]. Under conditions of uncompensated oxidative stress, NPD1 is made on demand from DHA when disruptors of homeostasis evolve, and the initial inflammatory response needs to be modulated to protect RPE cell integrity. We found that neurotrophins (*e.g*., BDNF, NGF, PEDF) [49] are agonists for the synthesis of this mediator in RPE cells, that 15-lipoxygenase-1 (15-LOX-1) is the enzyme that catalyzes its synthesis, that it targets protein phosphatase 2 A (PP2A) to regulate anti-/pro-apoptotic proteins during RPE oxidative stress, and that it regulates proteostasis in RPE cells. We identified transcription of pro-inflammatory genes as a target of NPD1 and demonstrated that the CA1 hippocampal area from short-post mortem, early-stage AD patients display a 25-fold loss of NPD1 as well as of the enzyme for its synthesis. A central theme of our laboratory is to understand early responses to hypoxia, oxidative stress, inflammation and conditions that recapitulate neurodegenerations to gain insight into mechanisms. Our common thread of concepts has included homeostatic regulation, necessary proteins, epigenetic events, and bioactive lipid mediators.

We uncovered that NPD1 arrests apoptosis in RPE cells at the pre-mitochondrial level and is neuroprotective in brain ischemia-reperfusion [47] and in cellular models of AD [50]. Esterified-DHA from phospholipids is cleaved by phospholipase A2 (PLA2), releasing DHA followed by NPD1 synthesis. Then, we showed that NPD1 promotes downregulation of pro-inflammatory genes and pro-apoptotic Bcl-2 proteins and that it also enhanced the abundance of anti-apoptotic proteins to counteract Aβ-mediated neurotoxicity.

We then found increased NPD1 synthesis after DHA administration in a middle cerebral artery occlusion (MCAo) stroke model that, in turn, prompts selective neuronal cREL translocation followed by BIRC3 gene expression, resulting in neurological recovery [51]. Thus, cREL was translocated into the nucleus, suggesting that NPD1 acts through cREL-mediated BIRC3 transcriptional activation to exert its neuroprotection. These studies also included a cell model, where we found that when the cREL protein abundance increases, it leads to survival and a decrease in p65/RelA in response to NPD1 [51].

NPD1 is a stress/injury-response mediator made on demand that counteracts disruptions of cellular homeostasis and is an active participant in a well-concerted process that effectively modulates neuroinflammation. Our lab found DHA protective bioactivity in the retina and brain and identified specific molecular mechanisms targeted by NPD1. Following the early observation of ischemia-mediated released DHA [47], with our colleagues, we demonstrated that DHA induces cell survival, modulates neuroinflammation, and activates long-term restoration of synaptic circuits in models of epileptogenesis [44]. DHA improves recovery after experimental ischemic stroke and, through its conversion into NPD1, halts homeostasis disruptions and cell death signals. As a consequence of DHA administration in the ischemic stroke MCAo model, increased NPD1 synthesis prompts selective neuronal cREL translocation followed by BIRC3 expression, resulting in remarkable neurological recovery [51]. Thus, we demonstrated that cREL, in a neuronal-specific fashion, was translocated into the nucleus upon i.v. DHA after experimental ischemic stroke [52], suggesting that NPD1 produced by the conversion of systemically administered DHA acts through cREL-mediated BIRC3 transcriptional activation to elicit its neuroprotective bioactivity. In our cellular model, we also found that when the cREL protein increases, it leads to survival and a decrease in p65/RelA in response to NPD1 [51]. These observations help to further unravel the endogenous signaling that sustains cellular integrity, thus providing a greater understanding of these mechanisms that could lead to novel, precise therapeutics for neuroprotection.

**Fig. 1:**
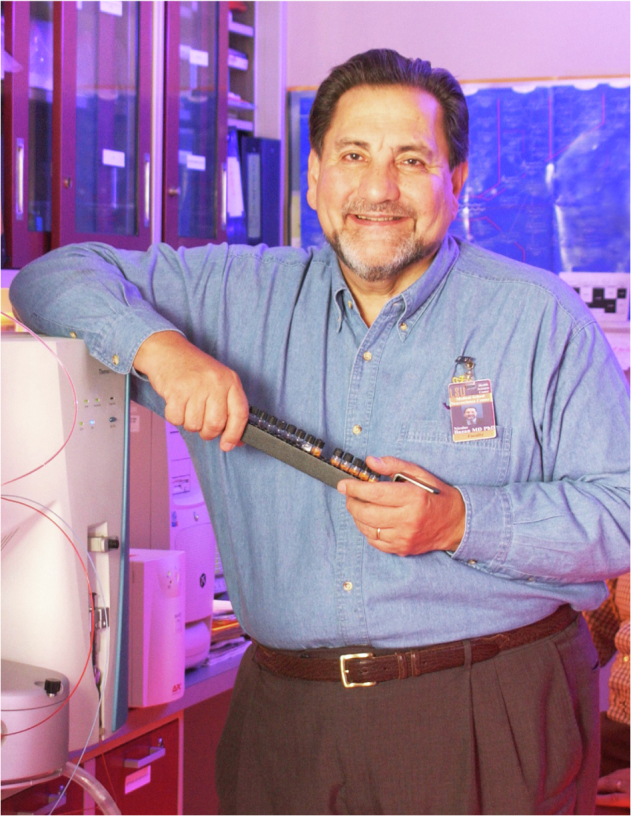
Nicolas G. Bazan, MD, PhD.

## CDDiscovery: we would like to know some more details on your work on elovanoids (ELVs), novel neuroprotective mediators in response to homeostatic disruptions

**Fig. 2:**
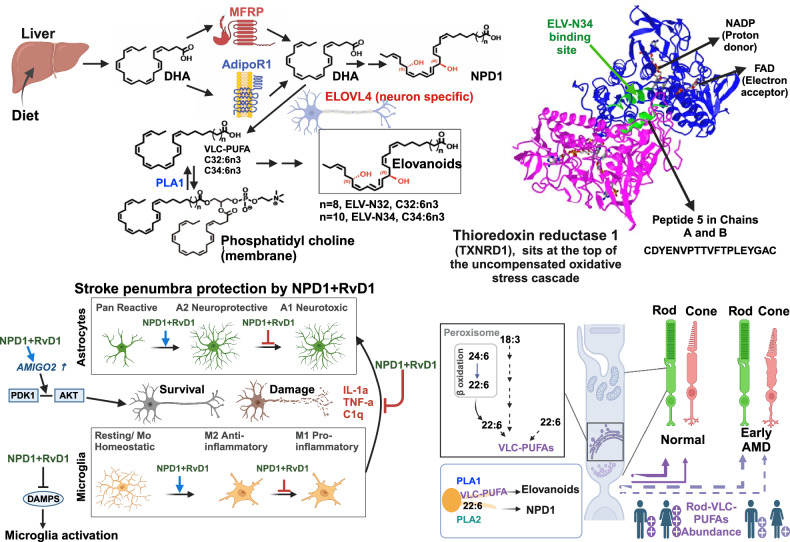
Neuroprotection signaling [53, 54], lipid precursors and pathways leading to Neuroprotectin D1 (NPD1) [47–49] and elovanoids (ELVs) [39, 41]. Uncompensated oxidative stress (UOS) targeted by ELV-34 [55], stroke penumbra protection [63], and age-related macular degeneration (AMD) [64–67] as an example of an age-related neurodegenerative disease.

We reported the finding of a new family of lipid messengers, for which we coined the name elovanoids (ELVs) [53, 54]. ELVs are set apart from all other lipid messengers, such as prostaglandins, leukotrienes, lipoxins, resolvins, and docosanoids, that are derived from 18C, 20C, and 22C-length fatty acid precursors. ELVs, on the other hand, are derived from 32C or 34C precursors with different properties. We reported the complete structures and stereochemistry of ELV-N32 (derived from 32:6,n-3) and ELV-N34 (derived from 34:6,n-3), the complete R/S configuration, and the Z/E geometry of the double bonds as generated in retinal cells and neurons. Our work showed that ELVs are cell-specific mediators necessary for neuroprotective signaling for cell integrity involving pathways that include ELV-N34 targeting *TXNRD1* to protect photoreceptor cell and RPE cell integrity [55] (see Fig. 2). Protein nitrosylation may be engaged in integrating responses to hypoxia and uncompensated oxidative stress (UOS) [56, 57]. This is a question for the future. ELVs also exert redundant modulation to Aβ-induced senescence gene programming and inflammaging [58].

Our ongoing quest in the fields of biology and medicine is, in a way, reflected as a response to one major challenge to civilization: the growing incidence in the loss of sight and cognition due to increased life expectancy and other factors [59]. The significance of lipids in retina function [31] now gets expanded by the identification of specific protective mediators.

Our ideas are synergized with a rise in neuronal survival and photoreceptor failure, as reflected mainly by and AD and AMD. Although age is the main risk factor, not everyone develops these diseases during aging. Thus, we posed the following questions: why can the latency period last for decades without disease manifestation, for example, in inherited familial forms of AD and in retinal degenerations, including AMD? And does a cell-specific initial response(s) counteract the consequences of mutation/polymorphism expression [59]? Are some initial responses triggered by hypoxia? There are many factors involved, including developmentally expressed genes. One goal is to decipher the molecular logic that sustains neuronal survival by uncovering molecular principles (transcriptional signatures) governed by the activation of hypoxia-mediated synthesis of NPD1 and ELVs.

We are unraveling issues, including the decision-making process involved in the storage specificity/retrieval of lipid mediators and the molecular sensors in the early stages of neurodegenerations. Several enzymatic systems are engaged in configuring lipid membrane organization [60–62]. The control of key neuroprotective and pro-homeostatic events by ELVs and NPD1 is a largely unappreciated mode of cell regulation that underlies resiliency.

Therefore, hypoxia sets specific beneficial effects in motion. It remains to be defined if the HIF-transcriptional regulation, the effects on browning adipose tissue, and the formation of neuroprotective lipid mediators are coordinated or if they are independent events of hypoxia to sustain cell functional integrity. Likely, they are regulated at multiple levels to fulfill the critical role of protection of cognition and sight, comprising a well-orchestrated redundancy and residency signaling. We are currently actively defining how elovanoids and other molecules target synaptic deconstruction by inducing protective cell phenotypes that modulate mitochondria function and uncompensated oxidative stress in stroke, TBI in models of neurodegenerative diseases, well as in Alzheimer's disease patients. Thus, advances in our understanding of these mechanisms would open avenues for therapeutic development.
